# Palliative nurses’ experiences of alleviating suffering and preserving dignity

**DOI:** 10.1177/09697330251326235

**Published:** 2025-03-20

**Authors:** Erika Storm, Elisabeth Bergdahl, Oscar Tranvåg, Yulia Korzhina, Cecilia Linnanen, Heidi Blomqvist, Jessica Hemberg

**Affiliations:** 1040 Åbo Akademi University; 6233 Örebro University; 1657 Western Norway University of Applied Sciences; 155272 Oslo University Hospital; 1040 Åbo Akademi University

**Keywords:** Caring, suffering, dignity, ethical sensitivity, compassion, palliative homecare

## Abstract

**Background:**

Most patients in need of palliative care remain in their homes, thus great focus should be placed on the creation of functional palliative homecare. Suffering through an often multifaceted illness and contemplating one’s death can contribute to the loss of one’s sense of dignity, and the preservation of patient dignity is a major challenge for health professionals worldwide.

**Aim:**

The aim of the study was to explore and describe nurses’ experiences of caring qualities alleviating suffering and preserving the dignity of patients in need of palliative homecare.

**Research design:**

A qualitative exploratory study. In-depth semi-structured interviews as data collection method, and the qualitative content analysis of Graneheim and Lundman for data analysis. The theoretical perspective was based on Eriksson’s caritative caring theory.

**Participants and research context:**

A total of nine nurses with extensive work experience from a palliative homecare context participated in the study.

**Ethical considerations:**

The study was conducted in accordance with the criteria set forth by the Finnish National Board on Research Integrity TENK. Research permission was granted and participants gave their written informed consent to participate in the study.

**Findings:**

One main theme and three subthemes were found. The main theme was: Being there for the other alleviates suffering while shaping and reshaping dignity preservation in a process. The three subthemes were: (1) Being a sensitive and compassionate witness who becomes responsible, (2) Having compliance, courage, and perception in a deep presence, (3) Being calm and patient while having time for conducting skilled practical knowledge.

**Conclusions:**

Certain caring qualities are important in the dignity-preserving care of people in need of palliative homecare, and person-centeredness plays a central role in alleviating suffering. Deep and trusting caring relationships and nurses’ ability to customize the care being provided are significant in alleviating patient suffering and preserving dignity.

## Introduction

According to the World Health Organization,^1^ the need for palliative care continues to grow on a global scale as chronic diseases become more common and life expectancy increases. More than a quarter of the world’s total population will be aged 65 or over by 2050, with the largest increase in percentage terms occurring in those over 85 years. Most patients in need of palliative care remain in their homes, thus great focus should be placed on the creation of functional palliative homecare.^1^ An organizational shift in palliative care, from being given on wards to taking place in the home with the support of a palliative care team, has resulted in the increased involvement of family in the care process.^2^ The support and treatment provided by healthcare professionals to patients and their families has a major impact on the patient’s overall well-being and their perception of own health and well-being.^3,4^ Deep, co-creative caring relationships are necessary to address ethical and existential issues that may arise at the end of life and to meet patients’ and family members’ expectations of palliative care.^5,6^

## Background

The core of palliative care is the promotion of quality of life and the provision of support to family caregivers.^1^ Aimed at patients with incurable diagnoses, for example, cancer, severe end-stage chronic disease, or extreme old-age frailty, palliative care includes a focus on measures that improve quality of life.^1,7^ Palliative care is team-based and can be provided over shorter periods or up to several years.^1,8^ Early adherence has been shown to increase patient life expectancy, improve patient satisfaction with care and quality of life, and reduce hospital costs.^8^ Researchers has also documented a clear reduction in emergency hospitalizations among patients receiving palliative care at home and an increase in the number of patients dying at home rather than in hospital wards.^8^

An important concept in palliative care, person-centeredness entails a holistic approach to the patient that enables a safe caring relationship, in which the patient and their family are involved in decision-making.^1,9,10^ The patient’s wishes, progression and prognosis of their disease, and the various symptoms that may arise are taken into consideration when creating a patient palliative care plan.^7,10^ Despite the diagnosis of serious disease, it is important that the patient can focus on continuing to live as active and meaningful a life as possible during their remaining life span.^8^

In the Nordic countries, interdisciplinary care teams are standard, and nurses, doctors, social workers, physiotherapists, and occupational therapists comprise multiprofessional teams.^5,11^ In many cases, such teams can also include dieticians and counselors. Outside of the Nordic countries, palliative care at home can be organized as either general or specialized palliative homecare, with specialized palliative homecare including round-the-clock care and direct access to a place on a ward as needed.^12^

Maintaining a sense of dignity is central to each person’s health and quality of life.^13^ Those in need of palliative care can often experience a decline in functions and roles, which can negatively impact their social and existential self-image^14^ and lead to suffering. In a palliative context, suffering can be viewed as a result of patient exhaustion due to the strain of coping with illness.^15^ The relief of suffering is a key goal within palliative care.^16^ Moreover, suffering through an often multifaceted illness and contemplating one’s death can contribute to the loss of one’s sense of dignity, and the preservation of patient dignity is a major challenge for healthcare professionals worldwide.^14^ Dignity preservation entails individualized care, the restoration of control to the patient, respect, advocacy, and sensitive listening^17^ and is a central part of patient care, for example, for patients with cancer who are cared for at home.^18^ Illness-related changes can potentially diminish dignity for patients, and the active presence of family members play an important role in preserving dignity because family members can provide the patient with a sense of pride and identity.^19^ Also health professionals can contribute to the preservation of dignity through professional knowledge, responsibility, and reflection as prerequisites that enable dignity preservation in care.^17^ Still, further research is needed, especially within palliative homecare,^18,20,21^ because dignity is central to patients’ perceived quality of life.^5^ Ethical sensitivity can thus be considered an essential concept in caring. Ethical sensitivity can be defined as the ability to make decisions with intelligence and compassion despite uncertainty in care situations, the ability to trust one’s own understanding of the ethical codes of conduct and one’s clinical expertise, and the courage to act.^22^ Ethical sensitivity can also be defined as an ability that a carer possesses through which they strive to understand and respond compassionately to the person in need of care. Ethical sensitivity can even be linked to a carer’s intuition about others’ emotional states or a carer’s ability to be “touched” by others or identify with others’ pain.^5^

Compassion is also a key concept in the preservation of dignity. As a concept, compassion can be used to describe how people are moved by the situation of others in such a way that they want to do something concrete for the other.^23^ Compassion can even be described as suffering with someone else and as the source of true caring, an ethical act in which the carer has the courage to take responsibility for the patient.^3^

## Theoretical perspective

The theoretical perspective was based on Eriksson’s caritative caring theory.^3,24^ The core of this theory is the alleviation of patient suffering and the preservation of dignity,^3,24^ which is in line with the overall goals of palliative care.^14,16^ Being in health entails that the patient is allowed to be in their own context, together with their family, and allowed to tend, play, and learn, that is, engage in a form of natural care. Optimal natural care entails that the patient, through own actions and in interaction with loved ones, creates a sense of bodily well-being, trust, and satisfaction, and thereby an opportunity to develop toward a higher level of integration. During this process, the person can experience faith, hope, and love, all of which together constitute the prerequisites for healing, that is, integration and health. Being human means being a unity of body, soul, and spirit.

Suffering is the struggle between good and evil, between suffering and desire.^3^ Human suffering can be recognized by sharing it with someone else, whether abstractly or concretely. Relieving a patient’s suffering entails that a carer does not violate the patient’s dignity and does not condemn or abuse their power: they care. Small acts can help preserve the patient’s dignity, for example, showing reverence for the patient, offering to wash their hands or comb their hair. Such acts can re-energize and give the patient a sense of their value as a human being. A kind glimpse, look, or word or other gesture that expresses the honest compassion of the nurse can alleviate even the most difficult suffering for a moment.^3^

## Aim

The aim of the study was to explore and describe nurses’ experiences of caring qualities alleviating suffering and preserving the dignity of patients in need of palliative homecare.

## Research design

A qualitative exploratory design was used.^25^ Data were collected using in-depth semi-structured interviews^26^ with nurses working in palliative homecare in Finland. Qualitative content analysis according to Graneheim and Lundman^27^ was used to analyze the data.

### Research context, participants, and recruitment

A purposive sample^28^ was chosen, consisting of nine nurses (females, aged 28–64) with extensive work experience (between 5 and 40 years) from a palliative homecare context (four in special palliative homecare, five in general palliative homecare). A flyer with information about the study was sent to head nurses in four different homecare districts responsible for Swedish-language speaking patients in Finland. The head nurses were asked to pass on the flyer to all nurses within the organization. Those interested in participation were asked to contact the first researcher by email to express their interest in being interviewed.

### Data collection

Nine in-depth semi-structured interviews^26^ were conducted between September 2022 to January 2023, lasting between 35 and 80 minutes each. An interview guide developed for the study was used. The interview guide was developed in relation to the study’s theoretical perspective and thus encompassed a special focus on the exploration of the themes of dignity and patient suffering, which are core perspectives in Eriksson’s caritative caring theory. Following two test interviews, some of the questions in the interview guide were reformulated to increase clarity. An opportunity was created during each interview for the interviewer to follow-up on any topic(s) that the interviewee(s) considered important. The interviews were conducted through a physical meeting between the first researcher and each individual research subject and were recorded digitally and transcribed verbatim. Data collection was ended after nine participant interviews because we experienced that no new empirical data were generated during the last few interviews.

### Data analysis

Qualitative content analysis according to Graneheim and Lundman^27^ was used to analyze the data. Initially, the interview transcriptions were read in their entirety several times. Text relevant to nurses’ experiences of caring qualities was extracted and compiled. Meaning units were thereafter identified, coded, and sorted into themes. An iterative process was used during interpretation, where the whole was compared against the parts and the parts then again compared against the whole. The focus of the analysis lay initially on describing and then on interpreting. During analysis we employed a particular focus on the latent meaning of the text and the underlying meaning extracted from the themes, that is, the latent content, which was formulated into a main theme. Following reflection and discussion, we then together agreed on the final main theme, subthemes, and overarching theme.

### Ethical considerations

The study was conducted in accordance with the criteria set forth by the Finnish National Board on Research Integrity TENK.^29^ A research ethics permit was granted (December 8, 2022) by the university where most of the researchers are based. Permission to conduct the research was also obtained from the municipal well-being service county (March 1, 2023) in Finland where the interviews were conducted. Prior to the interviews, the participants were informed about the purpose of the study and signed an informed consent form that included information about that participation in the study was voluntary. The participants were informed of their right to withdraw from the study without stating a reason, and data were managed with respect to the anonymity and confidentiality of all participants. All interviews were anonymized during transcription and securely stored.

## Findings

**Figure 1.**
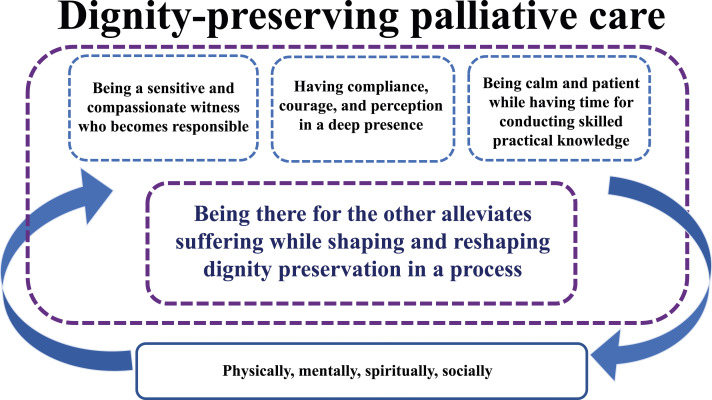
Overview of the findings.

One main theme and three subthemes were found. The main theme was: Being there for the other alleviates suffering while shaping and reshaping dignity preservation in a process. The three subthemes were: (1) Being a sensitive and compassionate witness who becomes responsible, (2) Having compliance, courage, and perception in a deep presence, (3) Being calm and patient while having time for conducting skilled practical knowledge. For an overview of the findings, see Figure 1.

The overarching theme of *Being there for the other relieves suffering while shaping and reshaping dignity preservation in a process* showed that caring qualities in the caring relationship shape and recreate caring acts in the moment. This entails a nurse being genuinely there for the other, which can create and recreate dignity for patients in need of palliative homecare. To alleviate patients’ suffering in palliative homecare, nurses should be sensitive and compassionate witnesses who become responsible. It is also important that nurses have compliance, courage, and perception in a deep presence, and that they are calm and patient while having time to conduct skilled practical knowledge.

### Being a sensitive and compassionate witness who becomes responsible

Nurses can alleviate suffering in the palliative homecare context by being sensitive and compassionate, that is, being open to each patient’s indications and expressions of suffering and having the willingness and courage to respond to these, showing benevolence, and seeing and listening to the suffering of the other. Accordingly, being sensitive as a nurse is a prerequisite for being compassionate toward the patient. When nurses see and sense the suffering patient in palliative homecare, they each become a witness who is responsible. According to the study participants, alleviating suffering in palliative homecare and preserving dignity can be achieved through certain acts of caring. For example, patient dignity can be strengthened through the caring act of compassion, which entails showing that you wish the patient well. *That you as a palliative carer want [to wish others] well. I see you, and I wish you well. It is the key word, otherwise you should not go into palliative care if you do not have the compassion and sympathy that it entails, that I wish you well and I want to help you* (P5). Nurses’ ability to show benevolence and compassion can take various forms.

Compassion is shown both through action and through discussion. Sympathy is shown more through that you care, you can tell me what you want me to do and I will do it—but that depth, that little extra that you really want the patient to have... with compassion you want to do all you can, and use all your knowledge for the patient’s best (P5).

The participants noted that they sought to acknowledge the patient’s and the patient’s family’s current situation, for example, by showing interest in the patient’s life story and thereby acknowledging the patient as a valuable person. “*I think surely that it is that, that you see them... you talk about their everyday life and let them do [different] things, you do not take anything away from them. You talk about what they have worked with…*(P1). Asking follow-up questions was a way of conveying compassion, and they addressed the importance of how nurses’ body language can convey a willingness to listen and help the patient. *Firstly, nurses must listen… not just listen but actively listen, and confirm with the patient that did I understand you correctly now, you mean like this?”* (P7). The participants perceived that a caring relationship can enable a compassionate encounter. Nurses should be perceptive and responsive to the moments when they notice that patients wish to discuss something. If the patient indicates a need to connect and talk, it is important that nurses make eye contact and show their openness to engaging in discussion. *I would say that is the most important thing, to listen and connect and give them time, to actually not be afraid of those questions*... (P4). The participants also mentioned presence as a fellow human being as being important. *Even if the nurse does not have any answers to give, it probably still helps the patient to see that there is a human being who listens and so on, although you may not always be able to give concrete advice or so, but just the fact that someone is listening [and is there]* (P3).

During conversation, nurses should seek to interpret “between the lines” of what is said and show a willingness to understand. For example, nurses might initially ask questions to carefully find out how the patient wants things to be done. The patient’s suffering must be recognized, and nurses therefore should listen to both what is said and what is left unsaid. *Most important is that you listen, that you actually sense what they are saying [even if they are not speaking]* (P2). This also applies to situations where the patient may be saying one thing but indicating something else with, for example, their eyes. *[Patients] should not feel forced but instead it should [come naturally], then you become close to them* (P7).

Attempts to alleviate the patient’s suffering in palliative homecare can take various forms according to the participants. This can include not only words and actions but also nurses’ ability to be in the moment with the patient, who is in need of dignity-preserving care. *That you actually are present, that you are in the moment. A seriously ill patient notices surely immediately if you are not there, even if you are there [physically]* (P2). Respecting the patient and trying to fulfill their wishes creates security. *It can of course be very little that is needed, even if you do not say anything... It is not always the words that matter but that you are there, you show that you are there if it is needed* (P9). Nurses’ mere presence can ease the patient’s situation and provide the patient a moment of rest from their suffering.

The participants described humility as the cornerstone of palliative homecare and the foundation for creating trust. A deep caring relationship is needed for successfully mediating compassion to the patient. A strong caring relationship also has a great impact on nurses’ perceptions of their ability to communicate compassion with both the patient and the patient’s family. Bringing the patient and family closer together while caring for the patient deepens the caring relationship, which can enable better end-of-life care. *Compassion is so much more comprehensive. I feel their grief, frustration...more personally, through that I have become closer to the patient* (P7). Concrete care activities can also lead to a deepening of the caring relationship; the patient can begin to trust their nurse or the patient’s family can sense the nurse’s benevolence and compassion. *There was a [patient] who we cared for until the last day in the home. ... [I] picked up a day cream that she had ... an expensive day cream that we put ... I have no idea if she knew what we were doing ... [but] I saw that the husband was very touched* (P5).

One of the participants perceived that it was important that nurses be able to share all the patient’s feelings. *You [feel for] the family. And surely it happens that I also can cry when we talk even though you are supposed to be professional... sometimes it is not possible, you become touched* (P1). According to the participants, showing emotions is a way of conveying compassion. *...if someone laughs, I laugh, if someone cries, I cry too. ...you do not have to have that facade in all situations* (P2). The participants also described how nurses’ ability to show emotion, understanding, and compassion to patients without suffering themselves was an important part of professional palliative homecare. However, the participants emphasized that nurses must be able to maintain their professionalism even when they are deeply affected so that they themselves do not need to be comforted, that is, they should maintain the strength needed to comfort the patient and the patient’s family. *It is up to relatives to grieve... the staff should not grieve because they are professionals, they should help the family to be able to grieve* (P3).

### Having compliance, courage, and perception in a deep presence

To alleviate suffering in the palliative homecare context, nurses should demonstrate compliance and have courage and perception. Sensitivity to the patient’s physical, medical, and psychosocial needs is essential to achieve dignified and appropriate palliative homecare. The participants also perceived that the ability to let the patient take the lead in the caring situation is a key element. *I accept each patient on their terms, that I do not come in and judge but instead am sensitive and get to know them first. It is so important... that you do everything their way* (P1). Waiting with patience and being responsive can, according to the participants, preserve the patient’s dignity, for example, if the patient disagrees with how a wound is being managed or what nurses are allowed to do during visits. It is vital that nurses balance closeness and distance, and it is through perception that nurses understand when to offer help or when to let the patient do something themselves. Nurses should convey that they are there if the patient needs something, even if the patient has previously declined offers of help. The participants noted that boundaries must sometimes be drawn because compliance with the patient’s wishes can at times be challenging, for example, regarding where the patient should spend their last days of life—at home or on a ward. Nurses’ sensitivity, ability, and courage to notice and acknowledge the nuances and wording of what is said plays a major role in ensuring that the patient’s wishes are honored. *Then [the last days of life], if ever, it is important that everything that happens, happens on the patient’s terms* (P9).

The participants maintained that it was important to be sensitive and responsive in the moment in palliative homecare, for example, to understand when an existential discussion is appropriate. One participant described this as a “door” that opens during a discussion: that one must have the courage to “cross the threshold” together with the patient when the patient is ready because the “door” can close again quickly.

Some days you see when that door opens, when you can ask …the difficult questions, when the person is open to them—it is not many minutes, sometimes just seconds before it closes, and then you must accept that today we do not discuss this. ...[We] laugh ... as much as we perhaps cry! You cannot be down there in the dark all the time but instead you must be lifted up sometimes...(P7).

Nurses can even sometimes “lift” the patient out of their suffering by providing a metaphorical “light against the darkness.” *I think many times…not so many visitors, and those who come start to cry…you need a little encouragement to come back to life* (P4).

The participants described situations where nurses must bear ethical responsibility. For example, if the interpersonal relationship between a nurse and patient is not quite right the nurse should seek to transfer the care of that patient to another nurse who is better suited to the patient. A participant stated that sensitivity can help them meet the patient’s unspoken expectations: their personal preferences or how they wish to be cared for. *For example... a motorized bed, I do not say hospital bed...I…talk about the benefits…but I cannot come in and say now we have to do this... It is a maturation process [for the patient]* (P7).

Presence in palliative homecare refers to nurses’ ability to be in the moment with and have the courage to be present for the patient, to share feelings and thoughts, which can deepen the caring relationship and enable even better care at the end of life. Sometimes helping the patient with a minor concern, listening to and having an open and honest conversation with the patient, or talking to the patient’s family can lead to a deepened caring relationship. The participants highlighted that it was crucial that nurses both give and receive and dare to be themselves. Mutual trust can also be built when nurses do more than what is strictly (task-related) needed or make things easier for the patient or the patient’s family. A strong caring relationship facilitates the sharing of humor even in dark situations, which can provide great relief. The participants also perceived that daring to face existential suffering through the act of caring presence was necessary, because nurses in palliative homecare encounter complicated family relationships and questions about death, the afterlife, and how existential suffering can be alleviated. *I think that even if the nurse does not have any answers to give, it probably still helps the patient to see that it is a person who is listening… even though you perhaps cannot always receive concrete advice.* (P3). Nurses can demonstrate through eye contact that they dare meet the patient and the patient’s family in such matters and “stay” in the discussion until the patient chooses to change the topic.

The participants emphasized that nurses should demonstrate sensitivity and courage concerning the ongoing grieving process that the palliative homecare patient whose life is about to end experiences. *That person is grieving all the time, they are grieving their body…that…they are not going to be around much longer…not going to see children or grandchildren.* (P7). Nurses should therefore be humble in the face of the patient’s impending death and be the patient’s companion. *I experience caring with compassion as like that you walk alongside the patient, that you accompany them* (P3). The participants also described the importance of encouraging the patient to retain their faith in the future, despite their illness.

…you might think that the patient only thinks about their funeral ... But then …the patient might say “next summer, then we will probably go there or do that.” And then I always answer encouragingly that, “Yes, I think you should do that, it sounds nice!” And it is perfectly natural…to think that way (P7).

### Being calm and patient while having time for conducting skilled practical knowledge

Nurses can alleviate suffering in a palliative homecare context by being calm and patient while conducting skilled practical knowledge. Conversely, if nurses radiate stress or impatience a deeper presence cannot be achieved. The participants perceived that it is more likely that the patient is satisfied after a visit and feels that the nurse has been in genuine presence with them if nurses take the time to do small, extra things for the patient despite time pressures. *But I think that it probably has of course a lot to do with oneself as well, that if I am super stressed then probably I can of course still take the time to do those little things that make it still feel okay for the patient* (P9). If nurses convey a calm presence during their visits, patients can forgive the fact that some visits must be shorter or less comprehensive, for example, because of a lack of time, etc.

The participants also described how confusion regarding terminology or language can occur in a palliative homecare, primarily between doctors and the patient. For example, many participants stated that they have taken the time following a doctor’s visit to review the patient’s medical records with the patient and translate the material into another language or explain the terminology or what is meant. Confusion can arise if the involved parties have different mother tongues, and the patient or patient’s family may find it difficult to understand the medical terminology used in medical notes (epicrisis). Nurses may need to calmly and with patience help with the translation and/or reviewing of notes or a discharge summary, which in palliative care can encompass statements relevant to a poor prognosis or a prognosis of impending death. *I usually quickly book a visit when I know that they have been to the oncologist and received bad news, when I know that the text is written, I take the epicrisis with me and we read it together, in peace and quiet around the kitchen table* (P7).

The prerequisite for nurses’ being able to provide quality care is their possession of professional knowledge. Yet if nurses must focus more on attempting to manage the medical aspects of care than on conveying compassion in palliative homecare an imbalance between the care situation and the conveyance of compassion can arise. Still, a lack of knowledge can lead to nurses’ uncertainty or nervousness in the care situation, which may inhibit their ability to convey compassion to the patient. Nurses’ own sense of integrity regarding how they themselves would wish to be cared for can also act as an ethical compass and help preserve the patient’s dignity, according to the participants. For example, aids (lifting or toilet aids, incontinence materials, etc.) can be placed in the home so the patient is not constantly exposed to reminders of their medical condition. *You should not leave things like incontinence diapers and all that, so that the patient has to see all the time that they have to use all that stuff now* (P5).

In palliative homecare nurses sometimes are invited to a patient’s funeral, which can be interpreted as a sign of the family’s appreciation, and reverence for the other is central in such situations: *But I thank them and say that I will not come, I would have over a funeral a week if I were to go to everyone’s*... *But it is so important to some that they can invite me to the funeral*... (P1). Conversations with the bereaved after the patient has passed away are also central when it comes to the realization of person-centered care. Nurses should, according to the participants, provide the patient’s family with the opportunity to speak with the nurse in a calm manner about the circumstances surrounding the patient’s death.

## Discussion

The aim of the study was to explore and describe nurses’ experiences of caring qualities alleviating suffering and preserving the dignity of patients in need of palliative homecare. As seen in the main theme, being there for the other alleviates suffering while shaping and reshaping dignity preservation in a process, certain caring qualities in the caring relationship shape and reshape the caring actions in the moment. It is important in the palliative homecare context that nurses preserve the patient’s dignity.^30–32^ This includes actively working in a dignity-preserving manner that encompasses both the patient and the patient’s family in the caring relationship^3^ and where the preservation of quality of life is an essential element in palliative care. The primary, overarching focus of palliative homecare is the alleviation of the patient’s suffering and ensuring that the patient’s wishes and will are heeded and fulfilled to the utmost extent possible.^3,9^

We found that being sensitive and compassionate is essential in the palliative homecare context and that such behavior makes nurses courageous and responsible. When nurses see and sense the patient’s suffering, they become a witness and consequently responsible. Nurses seek to mediate compassion and help, which philosophically can be related to Katie Eriksson’s mantra of care ethics: *I was there, I saw, I witnessed, and I became responsible*.^33^ The meaning of palliative care is two-fold, because it consists partly of alleviating suffering and partly of conveying compassion to the patient.^34^ As seen in our findings, being sensitive and compassionate and showing benevolence can alleviate suffering in a palliative homecare context. To convey their feelings and needs, a dying patient must trust their nurse.^35^ We discerned that nurses can convey compassion when they demonstrate that they seek to help the patient in any way they can, which can be considered a cornerstone in the palliative homecare context with respect to strengthening the patient’s dignity. Nurses can demonstrate compassion to the patient through concrete actions, for example, words, or by being there and sharing the moment during the care process.^35–37^ A small, concrete caring activity can mean a lot, because it can build trust.

The nurses perceived that compassion constituted a caring quality on a deeper level, transcending mere concern or sympathy. Compassion allows nurses to feel the patient’s grief, bringing them closer to the patient and enabling them to better understand the patient’s perspective. Other researchers describe compassion as a fundamental part of caring, something that every caregiver has an obligation to embody, because a lack of compassion can make the patient feel worthless and without emotional support.^38^ It is of great importance that nurses build a deep, mutual, and trusting relationship with the patient and thereby convey compassion, confirming the value of each human being and showing interest in their life history. Eriksson^3^ describes compassion as a sensitivity to the pain or suffering of others. This description is in line with the findings of this study, where compassion was described as *feeling for and with the patient, but not suffering yourself*. This can be compared with Bergdahl,^39^ who notes how important it is that a trusting caring relationship exists between a caregiver and patient, where both parties give and receive knowledge to deepen the caring relationship. Still, compassion as a phenomenon within the palliative care context can also hold a negative connotation, for example, relevant to compassion fatigue.^40–44^ Compassion fatigue can be defined as a sense of fatigue and inability to feel compassion for the patient, which can manifest as not being able to provide emotional support to the patient or the patient’s family. Causes of compassion fatigue can be linked to nurses’ personality traits, insufficient resources, or poor support from management.^43^

We also found that having compliance, courage, and perception in a deep presence is an essential caring quality in palliative homecare. Eriksson^3^ describes compassion as the source of true caring, noting that it requires courage to take responsibility for another person and sacrifice something of oneself. Courage was also seen in this study—the participating nurses described how their ethical sensitivity formed their way of caring for patients in palliative homecare. For example, when they as nurses faced situations where they were required to rely on their professional knowledge, experience, or ethical compass, while explaining to and motivating the patient concerning the most appropriate care options and simultaneously taking the patient’s own will and wishes into account in the decision-making process.^45^ A mutual give-and-take is needed in palliative homecare throughout the care process, which can be compared to *co-creation*, a phenomenon that has been increasingly researched in palliative research.^5,6,18,45–49^

In line with previous studies^9,36,37^ we found that nurses would, to the extent possible, let the patient control the care act. Nurse responsiveness and that nurses sense what is unsaid while also asking the patient direct questions are important.^5^ From our findings we discerned that it is important that nurses read the patient’s mood and emotional state. Also, it takes courage for nurses to follow their inner compass and bear ethical responsibility, for example, if the nurse-patient interpersonal relationship is not right and the patient would benefit from having a different carer/nurse. Carers’ emotional baggage or preconceived notions about a patient can hinder the provision of dignity-preserving care.^50^

The nurses in this study described how they sought to provide palliative homecare with the utmost respect and goodwill for the patient, the patient’s family, and the patient’s life and medical situation, and noted that they adapted the care they provided everyday so that it would best benefit the patient. This is a central finding and in line with the concepts of *co-creation* and *person-centered care*.^5,6,9,36,37,45–47,49^

In accordance with our findings, caring requires a lot of sensitivity and humility. In palliative homecare, it is pivotal that nurses respond to and acknowledge the patient’s existential suffering and have the courage to “walk alongside” the patient, in genuine openness and compliance. Nurses should even have the ability to “lift” the patient from their suffering and create opportunities for the person to feel alive despite their serious illness.^51,52^ Eriksson^3^ states that suffering breeds a feeling of hopelessness and that it takes courage for caregivers to face this suffering and share the hopelessness. Not being left alone in suffering preserves the patient’s dignity, provides hope, and alleviates suffering.^3^ Humor can also comprise an important element in palliative homecare because it can give the patient a temporary respite from their suffering, providing there exists a deep nurse-patient caring relationship in which both parties feel trust.^53,54^

From our findings we also learned that being calm while having time for conducting skilled practical knowledge is a caring quality that can promote patient dignity. To alleviate suffering in a palliative homecare context, it is vital that nurses remain calm and perform their practical work with skilled knowledge. Stress hinders a deep presence with the patient and the patient’s family. The art of caregiving is needed for nurses to seize the moment when the patient wishes to talk and is open to receive caregiving.^3,22^

The nurses in this present study described how it can be important within palliative homecare that the patient not be constantly reminded of their illness, noting that aids and material should be stored separately from where the patient spends most of their time. Also, it is emphasized in previous research that supporting the patient in focusing on continuing to live as actively as possible during their remaining lifetime is important and that palliative care plays a major role for both the patient and the patient’s family in the search for a meaningful everyday life.^8^

We also found that alleviating suffering and preserving dignity in palliative homecare is linked to being calm and open in the meeting with the patient. Time is needed to create trust in the caring relationship.^3^ A trusting caring relationship can help alleviate the patient’s suffering and is essential in palliative homecare.^3,5,39,55^ Strong trust and sufficient time can, in accordance with our findings, enable nurses’ sharing of a moment in a deep presence with the patient, which can strengthen the patient’s sense of dignity. We also discerned that nurses can use their sense of integrity to preserve the patient’s dignity, for example, by creating boundaries. The study participants described various aspects that could threaten the patient’s sense of dignity, for example, inconsistent care conditions, a lack of time, or confusion related to language or (medical) terminology. Working to ensure that conditions like these do not affect the quality of palliative homecare can also be perceived as important measures that can contribute to alleviating suffering and preserving the dignity of patients in need of end-of-life care. The patient’s experiences of caring qualities alleviating suffering and preserving the dignity of patients in palliative homecare should be investigated in future research.

## Methodological considerations

To enhance study trustworthiness, we focused on strengthening research credibility, dependability, confirmability, transferability, and authenticity.^56,57^ Following test interviews, the interview guide’s questions were modified before the participants’ interviews were conducted. Credibility was sought by allowing two of the researchers to thoroughly read the empirical data.^56^ The data material was read to ensure that relevant aspects that fulfilled the study purpose were identified, and analyzed. All of the researchers reflected thoroughly on the findings and a transparent^58^ description of the systematic methodology used has been provided, to promote the study’s dependability and confirmability. The participants’ wide and varied range of experience of the research subject may strengthen study reliability because it provided a solid and broad basis for the study.^27^ Study authenticity was emphasized through the participants’ own shared perspectives and experiences.^57^ All participants were females, and the lack of gender variation may be perceived as a study limitation with an impact on the study findings and its transferability. The study results were not validated with the participants due to the study time frame, which can be considered a study limitation. By emphasizing a transparent research process,^58^ we believe that our findings could hold relevance in other palliative homecare settings.

## Conclusion

Certain caring qualities—benevolence, sensing and listening, as well as compliance, courage, and perception in a deep presence—are perceived by nurses as vital in dignity-preserving care for people in need of palliative homecare and person-centeredness plays a central role in alleviating patients’ suffering. Essential to the foundation for such an approach is nurses’ ability to see the patient as a valuable entity. Deep and trusting caring relationships between the nurse, the patient, and the patient’s family are also of great importance for the alleviation of suffering. Nurses’ ability to customize the care being provided to each unique patient and home environment is one of the most significant qualities in alleviating suffering and preserving the dignity of the patient in the palliative homecare context. Our findings contribute to improved understanding of nurses’ experiences and practice related to alleviating suffering and preserving dignity in palliative homecare, but this knowledge can also contribute as a knowledge base for other healthcare professionals who encounter palliative care patients in their work.
